# Ethyl 2-amino-7,7-dimethyl-2′,5-dioxo­spiro­[5,6,7,8-tetra­hydro-4*H*-chromene-4,3′(2′*H*)-1*H*-indole]-3-carboxyl­ate

**DOI:** 10.1107/S1600536809055809

**Published:** 2010-01-09

**Authors:** Jing Wang, Song-Lei Zhu

**Affiliations:** a Department of Chemistry, Xuzhou Medical College, Xuzhou 221004, People’s Republic of China

## Abstract

In the mol­ecule of the title compound, C_21_H_22_N_2_O_5_, the indole system and the spiro-pyran ring are almost planar [maximum deviations of 0.0447 (17) and 0.0781 (17) Å, respectively]; the dihedral angle between them is 84.6 (3)°. The remaining six-membered ring adopts a twisted conformation. Intra­molecular N—H⋯O hydrogen bonds occur. In the crystal structure, intera­molecular N—H⋯O and C—H⋯O hydrogen bonds link the mol­ecules.

## Related literature

For the indole nucleus, see: da Silva *et al.*, (2001 ▶). For the anti­bacterial and fungicidal activities of indoles, see: Joshi & Chand (1982 ▶). Spiro­oxindole ring systems are found in a number of alkaloids, see: Abdel-Rahman *et al.* (2004 ▶). For our work on the preparation of heterocyclic compounds involving indole derivatives, see: Zhu *et al.* (2007 ▶). For puckering parameters, see: Cremer & Pople (1975 ▶).
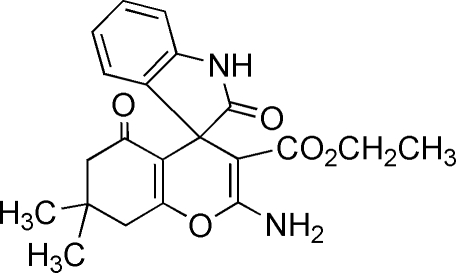


## Experimental

### 

#### Crystal data

C_21_H_22_N_2_O_5_
*M* *_r_* = 382.41
Monoclinic, 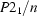

*a* = 8.4298 (14) Å
*b* = 11.6791 (17) Å
*c* = 19.024 (3) Å
β = 99.136 (4)°
*V* = 1849.2 (5) Å^3^
*Z* = 4
Mo *K*α radiation
μ = 0.10 mm^−1^
*T* = 153 K
0.50 × 0.35 × 0.12 mm

#### Data collection

Rigaku Mercury diffractometer
Absorption correction: multi-scan (Jacobson, 1998 ▶) *T*_min_ = 0.814, *T*_max_ = 0.988
17728 measured reflections
3374 independent reflections
2944 reflections with *I* > 2σ(*I*)
*R*_int_ = 0.041

#### Refinement

*R*[*F* ^2^ > 2σ(*F* ^2^)] = 0.049
*wR*(*F* ^2^) = 0.107
*S* = 1.15
3374 reflections
257 parameters
H-atom parameters constrained
Δρ_max_ = 0.20 e Å^−3^
Δρ_min_ = −0.23 e Å^−3^

### 

Data collection: *CrystalClear* (Rigaku/MSC, 2001 ▶); cell refinement: *CrystalClear*; data reduction: *CrystalStructure* (Rigaku/MSC, 2004 ▶); program(s) used to solve structure: *SHELXS97* (Sheldrick, 2008 ▶); program(s) used to refine structure: *SHELXL97* (Sheldrick, 2008 ▶); molecular graphics: *ORTEPII* (Johnson, 1976 ▶) and *PLATON* (Spek, 2009 ▶); software used to prepare material for publication: *SHELXL97* and *PLATON* (Spek, 2009 ▶).

## Supplementary Material

Crystal structure: contains datablocks global, I. DOI: 10.1107/S1600536809055809/ds2016sup1.cif

Structure factors: contains datablocks I. DOI: 10.1107/S1600536809055809/ds2016Isup2.hkl

Additional supplementary materials:  crystallographic information; 3D view; checkCIF report

## Figures and Tables

**Table 1:** Hydrogen-bond geometry (Å, °)

*D*—H⋯*A*	*D*—H	H⋯*A*	*D*⋯*A*	*D*—H⋯*A*
N1—H1*A*⋯O4	0.88	2.03	2.658 (2)	128
N1—H1*B*⋯O3^i^	0.88	1.92	2.794 (2)	175
N2—H2⋯O2^ii^	0.88	2.04	2.8435 (19)	152
C16—H16⋯O4^iii^	0.95	2.54	3.448 (2)	159

Symmetry codes: (i) 
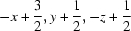
; (ii) 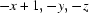; (iii) 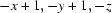.

**Fig. 1.**
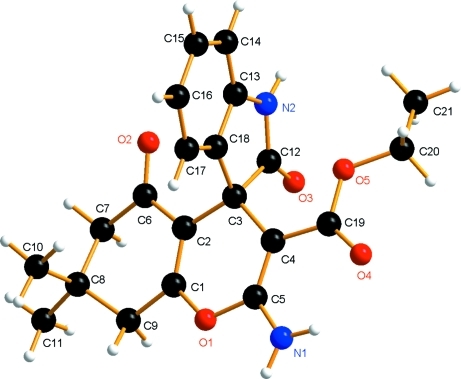
The molecular structure of the title molecule, with the atom-numbering scheme. Displacement ellipsoids are drawn at the 50% probability level.

**Fig. 2.**
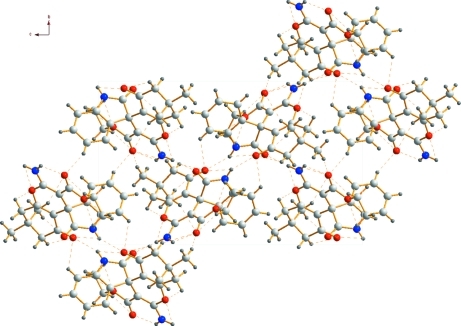
A packing diagram of (I). Hydrogen bonds are shown as dashed lines.

## supplementary crystallographic information

### Comment

The indole nucleus is the well known heterocycle (da Silva *et al.*,  2001). Compounds carrying the indole moiety exhibit antibacterial and fungicidal activities (Joshi & Chand, 1982). Spirooxindole ring systems are found in a number of alkaloids like horsifiline, spirotryprostatin and elacomine (Abdel-Rahman *et al.*, 2004). As a part of our programme devoted to the preparation of heterocyclic compounds involving indole derivatives (Zhu *et al.*, 2007), we have synthesized a series of spirooxindoles *via* reactions of substituted isatins together with malononitrile (or ethyl 2-cyanoacetate) and enaminones . We report herein the crystal structure of the title compound, (I).

In the molecule of (I), (Fig. 1),  the indole ring A (C3/C12/N2/C13-C18) and the spiro pyran ring B (O1/C1-C5), are planar. The dihedral angle between them is 84.6 (3)°. Ring C (C1/C2/C6-C9) adopts twisted conformation, with C7 and C8 deviating the C1/C2/C6/C9 plane by 0.1525 (18) and -0.4751 (18)Å, respectively. And the C1/C2/C6/C9 plane is oriented at a dihedral angle of 6.2 (2)° with the fused pyran ring B.

In the crystal structure, intermolecular and intramolecular N-H···O hydrogen bonds (N1-H1A···O4, N1-H1B···O3, N2-H2···O2. ) (Table 1) link the molecules (Fig. 2), in which they may be effective in the stabilization of the structure.

### Experimental

Compound (I) was prepared by one-pot reaction of isatin (2 mmol), ethyl 2-cyanoacetate (2 mmol) and 5,5-dimethylcyclohexane- 1,3-dione (2 mmol) in water (10 ml). The reaction was catalyzed by TEBAC (triethylbenzylammonium chloride, 1 mmol). After stirring at 333 K for 5 h, the reaction mixture was cooled and washed with small amount of ethanol. The crude product was filtered and single crystals of the title compound were obtained from ethanol solution by slow evaporation at room temperature (yield; 80%, m.p.  518-519 K). Spectroscopic analysis: IR (KBr, n, cm^-1^): 3364, 3241, 3187, 2955, 1690, 1613, 1520, 1474, 1304, 1227, 1165, 1057, 926, 748, 609, 556. ^1^H NMR (400 MHz, CDCl_3_): 7.48 (br s, 1H, NH), 7.14 (t, J = 7.2 Hz, 1H, ArH), 6,88-6.94 (m, 2H, ArH), 6.80 (t, J = 8.4 Hz, 1H, ArH), 6.49 (br s, 2H, NH_2_), 3.90-3.95 (m, 2H, CH_2_), 2.51-2.54 (m, 2H, CH_2_), 2.11-2.24 (m, 2H, CH_2_), 1.28 (t, J = 7.6 Hz, 3H, CH_3_), 1.10 (s, 3H, CH_3_), 1.01(s, 3H, CH_3_).

### Refinement

H atoms were positioned geometrically, with N-H = 0.88 Å (for NH) and C-H = 0.95 and 0.98 Å for aromatic and methyl H, respectivly and constrained to ride on their parent atoms with U_iso_(H) = xU_eq_(C,N), where x = 1.5 for methyl H and x = 1.2 for all other H atoms.

### Crystal data

**Table:**

C_21_H_22_N_2_O_5_	*F*(000) = 808
*M**_r_* = 382.41	*D*_x_ = 1.374 Mg m^−^^3^
Monoclinic, *P*2_1_/*n*	Melting point = 518–519 K
Hall symbol: -P 2yn	Mo *K*α radiation, λ = 0.71070 Å
*a* = 8.4298 (14) Å	Cell parameters from 6298 reflections
*b* = 11.6791 (17) Å	θ = 3.0–25.3°
*c* = 19.024 (3) Å	µ = 0.10 mm^−^^1^
β = 99.136 (4)°	*T* = 153 K
*V* = 1849.2 (5) Å^3^	Block, colorless
*Z* = 4	0.50 × 0.35 × 0.12 mm

### Data collection

**Table:**

Rigaku Mercury diffractometer	3374 independent reflections
Radiation source: fine-focus sealed tube	2944 reflections with *I* > 2σ(*I*)
graphite	*R*_int_ = 0.041
Detector resolution: 7.31 pixels mm^-1^	θ_max_ = 25.4°, θ_min_ = 3.0°
ω scans	*h* = −10→10
Absorption correction: multi-scan (Jacobson, 1998)	*k* = −14→13
*T*_min_ = 0.814, *T*_max_ = 0.988	*l* = −22→22
17728 measured reflections	

### Refinement

**Table:**

Refinement on *F*^2^	Primary atom site location: structure-invariant direct methods
Least-squares matrix: full	Secondary atom site location: difference Fourier map
*R*[*F*^2^ > 2σ(*F*^2^)] = 0.049	Hydrogen site location: inferred from neighbouring sites
*wR*(*F*^2^) = 0.107	H-atom parameters constrained
*S* = 1.15	*w* = 1/[σ^2^(*F*_o_^2^) + (0.040*P*)^2^ + 0.757*P*] where *P* = (*F*_o_^2^ + 2*F*_c_^2^)/3
3374 reflections	(Δ/σ)_max_ < 0.001
257 parameters	Δρ_max_ = 0.20 e Å^−^^3^
0 restraints	Δρ_min_ = −0.23 e Å^−^^3^

### Special details

**Table:**

Geometry. All esds (except the esd in the dihedral angle between two l.s. planes) are estimated using the full covariance matrix. The cell esds are taken into account individually in the estimation of esds in distances, angles and torsion angles; correlations between esds in cell parameters are only used when they are defined by crystal symmetry. An approximate (isotropic) treatment of cell esds is used for estimating esds involving l.s. planes.
Refinement. Refinement of F^2^ against ALL reflections. The weighted R-factor wR and goodness of fit S are based on F^2^, conventional R-factors R are based on F, with F set to zero for negative F^2^. The threshold expression of F^2^ > 2sigma(F^2^) is used only for calculating R-factors(gt) etc. and is not relevant to the choice of reflections for refinement. R-factors based on F^2^ are statistically about twice as large as those based on F, and R- factors based on ALL data will be even larger.

### Fractional atomic coordinates and isotropic or equivalent isotropic displacement parameters (Å^2^)

**Table:**

	*x*	*y*	*z*	*U*_iso_*/*U*_eq_	
O1	0.58254 (15)	0.33374 (11)	0.25244 (6)	0.0258 (3)	
O2	0.34545 (16)	0.04523 (12)	0.09660 (7)	0.0315 (3)	
O3	0.76243 (15)	0.05137 (11)	0.13053 (7)	0.0271 (3)	
O4	0.93311 (15)	0.42547 (11)	0.12924 (7)	0.0276 (3)	
O5	0.84779 (15)	0.28028 (12)	0.05510 (7)	0.0290 (3)	
N1	0.7795 (2)	0.45339 (14)	0.23971 (9)	0.0323 (4)	
H1A	0.8561	0.4825	0.2187	0.039*	
H1B	0.7603	0.4822	0.2803	0.039*	
N2	0.62096 (18)	0.09772 (13)	0.02113 (8)	0.0220 (3)	
H2	0.6506	0.0396	−0.0032	0.026*	
C1	0.4876 (2)	0.24020 (15)	0.23304 (9)	0.0213 (4)	
C2	0.4815 (2)	0.18729 (15)	0.17020 (9)	0.0200 (4)	
C3	0.5860 (2)	0.22220 (15)	0.11542 (9)	0.0200 (4)	
C4	0.7072 (2)	0.31183 (15)	0.14793 (9)	0.0209 (4)	
C5	0.6927 (2)	0.36589 (16)	0.21015 (9)	0.0229 (4)	
C6	0.3686 (2)	0.09070 (16)	0.15531 (10)	0.0226 (4)	
C7	0.2872 (2)	0.04575 (16)	0.21471 (10)	0.0255 (4)	
H7A	0.3577	−0.0125	0.2415	0.031*	
H7B	0.1864	0.0070	0.1936	0.031*	
C8	0.2471 (2)	0.13669 (16)	0.26702 (9)	0.0231 (4)	
C9	0.3984 (2)	0.20711 (17)	0.29162 (10)	0.0256 (4)	
H9A	0.3679	0.2775	0.3152	0.031*	
H9B	0.4710	0.1624	0.3275	0.031*	
C10	0.1122 (2)	0.21397 (18)	0.23047 (11)	0.0316 (5)	
H10A	0.1494	0.2558	0.1915	0.047*	
H10B	0.0818	0.2686	0.2651	0.047*	
H10C	0.0189	0.1670	0.2114	0.047*	
C11	0.1937 (2)	0.07742 (18)	0.33125 (10)	0.0309 (5)	
H11A	0.0951	0.0339	0.3155	0.046*	
H11B	0.1734	0.1352	0.3661	0.046*	
H11C	0.2784	0.0253	0.3532	0.046*	
C12	0.6698 (2)	0.11300 (15)	0.09176 (9)	0.0213 (4)	
C13	0.5176 (2)	0.18514 (15)	−0.00876 (9)	0.0215 (4)	
C14	0.4506 (2)	0.20069 (17)	−0.07895 (10)	0.0279 (5)	
H14	0.4678	0.1471	−0.1145	0.033*	
C15	0.3570 (2)	0.29760 (18)	−0.09573 (11)	0.0324 (5)	
H15	0.3084	0.3103	−0.1437	0.039*	
C16	0.3332 (2)	0.37610 (18)	−0.04389 (11)	0.0319 (5)	
H16	0.2705	0.4427	−0.0567	0.038*	
C17	0.4006 (2)	0.35815 (16)	0.02711 (10)	0.0261 (4)	
H17	0.3839	0.4116	0.0629	0.031*	
C18	0.4919 (2)	0.26158 (15)	0.04424 (9)	0.0206 (4)	
C19	0.8378 (2)	0.34677 (16)	0.11166 (9)	0.0226 (4)	
C20	0.9853 (2)	0.29242 (19)	0.01860 (11)	0.0353 (5)	
H20A	1.0827	0.3117	0.0529	0.042*	
H20B	0.9661	0.3540	−0.0175	0.042*	
C21	1.0062 (3)	0.1792 (2)	−0.01643 (12)	0.0434 (6)	
H21A	1.0244	0.1191	0.0199	0.065*	
H21B	1.0986	0.1833	−0.0417	0.065*	
H21C	0.9091	0.1615	−0.0503	0.065*	

### Atomic displacement parameters (Å^2^)

**Table:**

	*U*^11^	*U*^22^	*U*^33^	*U*^12^	*U*^13^	*U*^23^
O1	0.0258 (7)	0.0279 (7)	0.0249 (7)	−0.0071 (6)	0.0076 (5)	−0.0071 (6)
O2	0.0358 (8)	0.0318 (8)	0.0291 (8)	−0.0101 (6)	0.0120 (6)	−0.0115 (6)
O3	0.0269 (7)	0.0275 (7)	0.0271 (7)	0.0066 (6)	0.0047 (6)	0.0049 (6)
O4	0.0237 (7)	0.0265 (7)	0.0321 (7)	−0.0066 (6)	0.0031 (6)	0.0005 (6)
O5	0.0266 (7)	0.0351 (8)	0.0275 (7)	−0.0099 (6)	0.0117 (6)	−0.0045 (6)
N1	0.0331 (10)	0.0342 (10)	0.0308 (9)	−0.0124 (8)	0.0087 (7)	−0.0130 (8)
N2	0.0249 (8)	0.0209 (8)	0.0213 (8)	−0.0001 (7)	0.0074 (6)	−0.0028 (6)
C1	0.0190 (9)	0.0216 (10)	0.0230 (9)	−0.0001 (7)	0.0024 (7)	−0.0002 (8)
C2	0.0192 (9)	0.0202 (10)	0.0212 (9)	0.0000 (7)	0.0049 (7)	0.0006 (7)
C3	0.0193 (9)	0.0198 (9)	0.0211 (9)	−0.0006 (7)	0.0040 (7)	−0.0015 (7)
C4	0.0197 (9)	0.0204 (10)	0.0223 (9)	−0.0003 (7)	0.0020 (7)	0.0006 (7)
C5	0.0202 (9)	0.0247 (10)	0.0235 (10)	−0.0015 (8)	0.0024 (8)	−0.0001 (8)
C6	0.0214 (9)	0.0205 (10)	0.0266 (10)	0.0026 (8)	0.0061 (8)	−0.0024 (8)
C7	0.0255 (10)	0.0232 (10)	0.0291 (10)	−0.0012 (8)	0.0082 (8)	0.0007 (8)
C8	0.0212 (9)	0.0241 (10)	0.0253 (10)	0.0019 (8)	0.0072 (8)	0.0026 (8)
C9	0.0261 (10)	0.0285 (11)	0.0229 (10)	0.0035 (8)	0.0066 (8)	0.0001 (8)
C10	0.0237 (10)	0.0332 (12)	0.0383 (12)	0.0061 (9)	0.0062 (9)	0.0047 (9)
C11	0.0317 (11)	0.0330 (12)	0.0308 (11)	0.0012 (9)	0.0134 (9)	0.0045 (9)
C12	0.0207 (9)	0.0203 (10)	0.0242 (10)	−0.0039 (8)	0.0073 (8)	0.0009 (8)
C13	0.0189 (9)	0.0239 (10)	0.0220 (9)	−0.0050 (8)	0.0047 (7)	0.0005 (8)
C14	0.0280 (10)	0.0342 (11)	0.0218 (10)	−0.0088 (9)	0.0051 (8)	0.0005 (8)
C15	0.0267 (11)	0.0413 (13)	0.0270 (11)	−0.0059 (9)	−0.0021 (8)	0.0092 (9)
C16	0.0238 (10)	0.0308 (11)	0.0391 (12)	0.0014 (9)	−0.0005 (9)	0.0122 (9)
C17	0.0212 (10)	0.0239 (10)	0.0333 (11)	−0.0017 (8)	0.0045 (8)	0.0001 (8)
C18	0.0175 (9)	0.0209 (10)	0.0235 (9)	−0.0042 (7)	0.0039 (7)	0.0006 (7)
C19	0.0212 (9)	0.0235 (10)	0.0219 (9)	−0.0001 (8)	−0.0003 (7)	0.0032 (8)
C20	0.0305 (11)	0.0459 (13)	0.0333 (11)	−0.0100 (10)	0.0167 (9)	−0.0016 (10)
C21	0.0393 (13)	0.0514 (15)	0.0449 (13)	−0.0047 (11)	0.0229 (11)	−0.0058 (11)

### Geometric parameters (Å, °)

**Table:**

O1—C1	1.370 (2)	C8—C10	1.530 (3)
O1—C5	1.374 (2)	C8—C11	1.533 (3)
O2—C6	1.224 (2)	C9—H9A	0.9900
O3—C12	1.220 (2)	C9—H9B	0.9900
O4—C19	1.232 (2)	C10—H10A	0.9800
O5—C19	1.340 (2)	C10—H10B	0.9800
O5—C20	1.450 (2)	C10—H10C	0.9800
N1—C5	1.328 (2)	C11—H11A	0.9800
N1—H1A	0.8800	C11—H11B	0.9800
N1—H1B	0.8800	C11—H11C	0.9800
N2—C12	1.353 (2)	C13—C14	1.377 (3)
N2—C13	1.403 (2)	C13—C18	1.389 (3)
N2—H2	0.8800	C14—C15	1.387 (3)
C1—C2	1.339 (2)	C14—H14	0.9500
C1—C9	1.491 (3)	C15—C16	1.384 (3)
C2—C6	1.474 (3)	C15—H15	0.9500
C2—C3	1.523 (2)	C16—C17	1.396 (3)
C3—C4	1.524 (2)	C16—H16	0.9500
C3—C18	1.528 (2)	C17—C18	1.375 (3)
C3—C12	1.559 (3)	C17—H17	0.9500
C4—C5	1.364 (3)	C20—C21	1.503 (3)
C4—C19	1.448 (3)	C20—H20A	0.9900
C6—C7	1.507 (3)	C20—H20B	0.9900
C7—C8	1.530 (3)	C21—H21A	0.9800
C7—H7A	0.9900	C21—H21B	0.9800
C7—H7B	0.9900	C21—H21C	0.9800
C8—C9	1.527 (3)		
			
C1—O1—C5	118.72 (14)	C8—C10—H10B	109.5
C19—O5—C20	119.01 (15)	H10A—C10—H10B	109.5
C5—N1—H1A	120.0	C8—C10—H10C	109.5
C5—N1—H1B	120.0	H10A—C10—H10C	109.5
H1A—N1—H1B	120.0	H10B—C10—H10C	109.5
C12—N2—C13	112.20 (15)	C8—C11—H11A	109.5
C12—N2—H2	123.9	C8—C11—H11B	109.5
C13—N2—H2	123.9	H11A—C11—H11B	109.5
C2—C1—O1	123.13 (16)	C8—C11—H11C	109.5
C2—C1—C9	126.52 (17)	H11A—C11—H11C	109.5
O1—C1—C9	110.34 (15)	H11B—C11—H11C	109.5
C1—C2—C6	117.18 (16)	O3—C12—N2	125.94 (17)
C1—C2—C3	122.87 (16)	O3—C12—C3	125.73 (16)
C6—C2—C3	119.95 (15)	N2—C12—C3	108.32 (15)
C2—C3—C4	109.24 (14)	C14—C13—C18	122.13 (18)
C2—C3—C18	114.32 (14)	C14—C13—N2	128.45 (17)
C4—C3—C18	111.93 (14)	C18—C13—N2	109.39 (15)
C2—C3—C12	108.45 (14)	C13—C14—C15	117.47 (18)
C4—C3—C12	111.94 (14)	C13—C14—H14	121.3
C18—C3—C12	100.71 (14)	C15—C14—H14	121.3
C5—C4—C19	117.57 (16)	C16—C15—C14	121.19 (18)
C5—C4—C3	121.57 (16)	C16—C15—H15	119.4
C19—C4—C3	120.78 (15)	C14—C15—H15	119.4
N1—C5—C4	127.17 (17)	C15—C16—C17	120.47 (19)
N1—C5—O1	109.90 (15)	C15—C16—H16	119.8
C4—C5—O1	122.93 (16)	C17—C16—H16	119.8
O2—C6—C2	120.78 (16)	C18—C17—C16	118.64 (18)
O2—C6—C7	120.50 (17)	C18—C17—H17	120.7
C2—C6—C7	118.66 (16)	C16—C17—H17	120.7
C6—C7—C8	114.84 (16)	C17—C18—C13	120.07 (17)
C6—C7—H7A	108.6	C17—C18—C3	130.49 (17)
C8—C7—H7A	108.6	C13—C18—C3	109.33 (15)
C6—C7—H7B	108.6	O4—C19—O5	121.92 (17)
C8—C7—H7B	108.6	O4—C19—C4	126.41 (17)
H7A—C7—H7B	107.5	O5—C19—C4	111.66 (15)
C9—C8—C7	108.25 (15)	O5—C20—C21	106.56 (16)
C9—C8—C10	110.24 (16)	O5—C20—H20A	110.4
C7—C8—C10	109.82 (16)	C21—C20—H20A	110.4
C9—C8—C11	109.61 (15)	O5—C20—H20B	110.4
C7—C8—C11	109.16 (15)	C21—C20—H20B	110.4
C10—C8—C11	109.72 (15)	H20A—C20—H20B	108.6
C1—C9—C8	113.97 (15)	C20—C21—H21A	109.5
C1—C9—H9A	108.8	C20—C21—H21B	109.5
C8—C9—H9A	108.8	H21A—C21—H21B	109.5
C1—C9—H9B	108.8	C20—C21—H21C	109.5
C8—C9—H9B	108.8	H21A—C21—H21C	109.5
H9A—C9—H9B	107.7	H21B—C21—H21C	109.5
C8—C10—H10A	109.5		
			
C5—O1—C1—C2	−9.1 (3)	C10—C8—C9—C1	−76.1 (2)
C5—O1—C1—C9	169.84 (15)	C11—C8—C9—C1	163.06 (16)
O1—C1—C2—C6	−177.43 (15)	C13—N2—C12—O3	−178.32 (17)
C9—C1—C2—C6	3.8 (3)	C13—N2—C12—C3	2.29 (19)
O1—C1—C2—C3	3.2 (3)	C2—C3—C12—O3	−61.1 (2)
C9—C1—C2—C3	−175.58 (17)	C4—C3—C12—O3	59.5 (2)
C1—C2—C3—C4	7.4 (2)	C18—C3—C12—O3	178.58 (17)
C6—C2—C3—C4	−171.98 (15)	C2—C3—C12—N2	118.30 (15)
C1—C2—C3—C18	−118.89 (19)	C4—C3—C12—N2	−121.10 (16)
C6—C2—C3—C18	61.7 (2)	C18—C3—C12—N2	−2.03 (17)
C1—C2—C3—C12	129.65 (18)	C12—N2—C13—C14	176.80 (18)
C6—C2—C3—C12	−49.7 (2)	C12—N2—C13—C18	−1.6 (2)
C2—C3—C4—C5	−13.0 (2)	C18—C13—C14—C15	1.0 (3)
C18—C3—C4—C5	114.60 (19)	N2—C13—C14—C15	−177.16 (17)
C12—C3—C4—C5	−133.18 (18)	C13—C14—C15—C16	0.6 (3)
C2—C3—C4—C19	170.14 (16)	C14—C15—C16—C17	−1.3 (3)
C18—C3—C4—C19	−62.2 (2)	C15—C16—C17—C18	0.5 (3)
C12—C3—C4—C19	50.0 (2)	C16—C17—C18—C13	1.1 (3)
C19—C4—C5—N1	4.7 (3)	C16—C17—C18—C3	176.77 (18)
C3—C4—C5—N1	−172.18 (17)	C14—C13—C18—C17	−1.8 (3)
C19—C4—C5—O1	−174.29 (16)	N2—C13—C18—C17	176.64 (16)
C3—C4—C5—O1	8.8 (3)	C14—C13—C18—C3	−178.38 (16)
C1—O1—C5—N1	−176.23 (15)	N2—C13—C18—C3	0.1 (2)
C1—O1—C5—C4	2.9 (3)	C2—C3—C18—C17	69.0 (2)
C1—C2—C6—O2	173.11 (17)	C4—C3—C18—C17	−55.8 (2)
C3—C2—C6—O2	−7.5 (3)	C12—C3—C18—C17	−174.93 (18)
C1—C2—C6—C7	−9.4 (2)	C2—C3—C18—C13	−114.90 (17)
C3—C2—C6—C7	170.05 (16)	C4—C3—C18—C13	120.22 (16)
O2—C6—C7—C8	−147.85 (17)	C12—C3—C18—C13	1.13 (18)
C2—C6—C7—C8	34.6 (2)	C20—O5—C19—O4	7.4 (3)
C6—C7—C8—C9	−50.6 (2)	C20—O5—C19—C4	−171.23 (16)
C6—C7—C8—C10	69.8 (2)	C5—C4—C19—O4	−4.3 (3)
C6—C7—C8—C11	−169.86 (16)	C3—C4—C19—O4	172.61 (17)
C2—C1—C9—C8	−23.1 (3)	C5—C4—C19—O5	174.22 (16)
O1—C1—C9—C8	158.03 (15)	C3—C4—C19—O5	−8.8 (2)
C7—C8—C9—C1	44.1 (2)	C19—O5—C20—C21	154.05 (17)

### Hydrogen-bond geometry (Å, °)

**Table:**

*D*—H···*A*	*D*—H	H···*A*	*D*···*A*	*D*—H···*A*
N1—H1A···O4	0.88	2.03	2.658 (2)	128
N1—H1B···O3^i^	0.88	1.92	2.794 (2)	175
N2—H2···O2^ii^	0.88	2.04	2.8435 (19)	152
C16—H16···O4^iii^	0.95	2.54	3.448 (2)	159

Symmetry codes: (i) −*x*+3/2, *y*+1/2, −*z*+1/2; (ii) −*x*+1, −*y*, −*z*; (iii) −*x*+1, −*y*+1, −*z*.
